# Predicting the Risk of Sleep Disorders Using a Machine Learning–Based Simple Questionnaire: Development and Validation Study

**DOI:** 10.2196/46520

**Published:** 2023-09-21

**Authors:** Seokmin Ha, Su Jung Choi, Sujin Lee, Reinatt Hansel Wijaya, Jee Hyun Kim, Eun Yeon Joo, Jae Kyoung Kim

**Affiliations:** 1 Biomedical Mathematics Group Institute for Basic Science Daejeon Republic of Korea; 2 Department of Mathematical Sciences Korea Advanced Institute of Science and Technology Daejeon Republic of Korea; 3 Graduate School of Clinical Nursing Science Sungkyunkwan University Seoul Republic of Korea; 4 Department of Neurology Neuroscience Center, Samsung Medical Center Sungkyunkwan University School of Medicine Seoul Republic of Korea; 5 Department of Computer Science Korea Advanced Institute of Science and Technology Daejeon Republic of Korea; 6 Department of Neurology Ewha Womans University Seoul Hospital Ewha Womans University College of Medicine Seoul Republic of Korea

**Keywords:** obstructive sleep apnea, insomnia, comorbid insomnia and sleep apnea, polysomnography, questionnaires, risk prediction, XGBoost, machine learning, risk, sleep

## Abstract

**Background:**

Sleep disorders, such as obstructive sleep apnea (OSA), comorbid insomnia and sleep apnea (COMISA), and insomnia are common and can have serious health consequences. However, accurately diagnosing these conditions can be challenging as a result of the underrecognition of these diseases, the time-intensive nature of sleep monitoring necessary for a proper diagnosis, and patients’ hesitancy to undergo demanding and costly overnight polysomnography tests.

**Objective:**

We aim to develop a machine learning algorithm that can accurately predict the risk of OSA, COMISA, and insomnia with a simple set of questions, without the need for a polysomnography test.

**Methods:**

We applied extreme gradient boosting to the data from 2 medical centers (n=4257 from Samsung Medical Center and n=365 from Ewha Womans University Medical Center Seoul Hospital). Features were selected based on feature importance calculated by the Shapley additive explanations (SHAP) method. We applied extreme gradient boosting using selected features to develop a simple questionnaire predicting sleep disorders (SLEEPS). The accuracy of the algorithm was evaluated using the area under the receiver operating characteristics curve.

**Results:**

In total, 9 features were selected to construct SLEEPS. SLEEPS showed high accuracy, with an area under the receiver operating characteristics curve of greater than 0.897 for all 3 sleep disorders, and consistent performance across both sets of data. We found that the distinction between COMISA and OSA was critical for accurate prediction. A publicly accessible website was created based on the algorithm that provides predictions for the risk of the 3 sleep disorders and shows how the risk changes with changes in weight or age.

**Conclusions:**

SLEEPS has the potential to improve the diagnosis and treatment of sleep disorders by providing more accessibility and convenience. The creation of a publicly accessible website based on the algorithm provides a user-friendly tool for assessing the risk of OSA, COMISA, and insomnia.

## Introduction

Insomnia and obstructive sleep apnea (OSA) are two of the most common sleep disorders, affecting up to 40% and 46% of the general population, respectively [1-6]. Insomnia, a clinical sleep disorder characterized by difficulty falling asleep, staying asleep, or waking up too early, leads to daytime impairment, including fatigue, attention problems, mood changes, or impaired performance; it is estimated that over one-third of individuals with insomnia experience chronic symptoms [7,8]. Diagnosis of insomnia is typically based on clinical symptoms rather than sleep parameters measured by overnight polysomnography (PSG), although PSG can identify potential underlying causes of insomnia-related symptoms [9,10]. OSA is characterized by repeated episodes of partial or complete upper airway obstruction during sleep, which can lead to sleep fragmentation and poor sleep quality. In-laboratory overnight PSG is the gold standard for diagnosing OSA [11]. Both insomnia and OSA can lead to serious health problems such as cardiovascular, metabolic, and neurological disorders [2,3,12,13]. Patients with OSA often have trouble falling asleep or staying asleep. Thus, comorbid insomnia and sleep apnea (COMISA) is the most common co-occurring sleep disorder, which affects up to 42% of people globally [14]. COMISA is associated with increased medical and psychiatric morbidity and worse daytime functioning relative to each condition alone [15-20]. However, most people with COMISA do not recognize the possibility of accompanying OSA and do not undergo OSA evaluation [10,14,15]. Thus, it is clinically critical to differentiate patients with COMISA from insomnia patients without specific sleep disorders to cause insomnia symptoms, which requires the PSG test.

Despite the high prevalence of OSA and insomnia, these disorders often go unrecognized and untreated due to a lack of awareness [21-23]. This is due to the underrecognition of these diseases, the time-consuming nature of sleep monitoring required for an appropriate diagnosis [23,24], and patient reluctance to undergo cumbersome and expensive PSG tests. To address this issue, simple questionnaires have been developed to predict the risk of OSA or insomnia [25-35]. To the best of our knowledge, there is currently no single questionnaire available that can differentially diagnose OSA, COMISA, and insomnia. Although there are several screening tools used to screen these 3 disorders individually, such as STOP-BANG [25], the Berlin Questionnaire [26], the Insomnia Severity Index (ISI) [27], the Epworth Sleepiness Scale (ESS) [28], and the Pittsburgh Sleep Quality Index (PSQI) [29], an increasing number of questions need to be answered to detect and differentiate one sleep disorder from the others. Moreover, some of the surveys have overlapping parts, making such a process inconvenient. In addition, these questionnaires require observed breathing cessation [25,26], blood pressure measurements [30], and neck circumference measurements [28], which are difficult for the general public to measure. Furthermore, their prediction accuracy is not satisfactory (eg, area under the receiver operating characteristics curve [AUROC]<0.79) [34,35]. Hence, there is a growing demand in clinical settings for a single questionnaire that can accurately predict the risk of all 3 sleep disorders.

**Figure 1:**
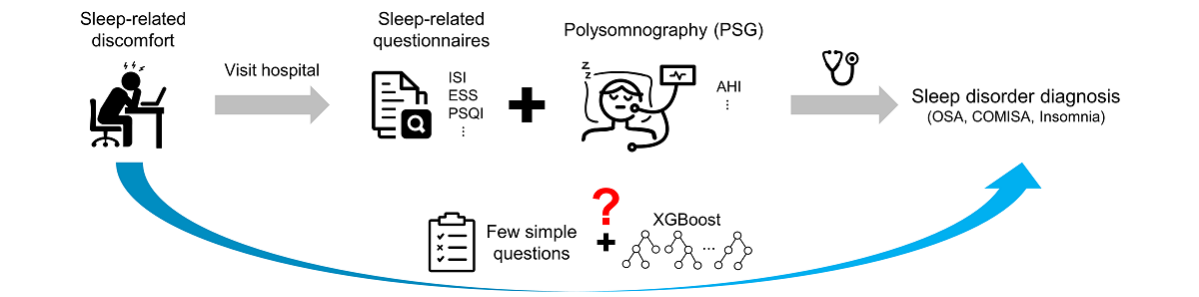
Approach overview. The diagnosis of sleep disorders typically involves a combination of questionnaires and polysomnography (PSG), which can be costly and cumbersome. Additionally, the underrecognition of sleep-related disease and the time-consuming nature of sleep monitoring required for an appropriate diagnosis contribute to the complexity of the process. Our aim is to simplify this process by predicting the risk of the most prevalent sleep disorders, OSA, COMISA, and insomnia, using simple questionnaires. AHI: Apnea-Hypopnea Index; COMISA: comorbid insomnia and sleep apnea; ISI: Insomnia Severity Index; ESS: Epworth Sleepiness Scale; OSA: obstructive sleep apnea; PSQI: Pittsburgh Sleep Quality Index; XGBoost: extreme gradient boosting.

Therefore, we aimed to develop a machine learning algorithm that can accurately predict the risk of OSA, COMISA, and insomnia using simple questions without needing PSG (Figure 1). We accomplished this by identifying simple 9 questions, with which extreme gradient boosting (XGBoost) [36] accurately predicted the risk of the 3 sleep disorders (AUROC>0.897). The model also showed consistent performance on an independent test set, indicating its universal applicability. Based on this algorithm, we created a publicly accessible website [37] providing predictions for the risk of the 3 sleep disorders. The website also provides information on how the risk changes with variations in the user’s weight or age, which is important for treatment planning. The use of this screening model for major sleep disorders may help more patients with occult sleep disorders receive early attention and management to prevent disease-related complications.

## Methods

### Approach Overview

The PSG data were collected from Samsung Medical Center (SMC, n=4257) and Ewha Womans University Medical Center Seoul Hospital (EUMCSH, n=365). We randomly divided the SMC data set into training and testing sets in a ratio of 8:2. In this paper, the training set (testing set) is referred to as the SMC training set (SMC testing set). We applied 3 XGBoost models to the SMC training set, classifying participants with OSA, COMISA, and insomnia, respectively, using items from sleep-related questionnaires and demographic characteristics as input features (Figure 1). To calculate the feature importance, the Shapley additive explanations (SHAP) [38] method is used. Based on computed feature importance, features with high importance were selected. Then, we made new XGBoost models predicting sleep disorders using only these selected features. The classification performance of the model was verified using the SMC testing set. The EUMCSH data set was used as an independent test set to check the universality of the model.

### Data Collection

#### Ethics Approval

The study protocol was reviewed and approved by the Institutional Review Board of the SMC (approval 2022-07-003) and was conducted in accordance with the principles of the Declaration of Helsinki. Participant informed consent was waived due to the retrospective nature of the study.

#### Inclusion and Exclusion Criteria

The data for this study were collected from participants who underwent PSG at the SMC sleep clinic from December 2014 to August 2021 and at the EUMCSH from January 2020 to June 2022. The study excluded participants who met any of the following criteria: (1) they were 18 years old or younger; (2) they were not fluent in Korean; (3) they worked in shifts; (4) they experienced significant medical, mental, or neurological disorders; or (5) they experienced other sleep disorders, such as narcolepsy, rapid eye movement sleep behavior disorder, restless legs syndrome, or periodic limb movement disorder confirmed by PSG with a total periodic limb movement index of 15 events per hour or higher and a movement arousal index equal to or greater than 5 events per hour. A total of 4257 participants’ data were collected from the SMC, and 365 participants’ data was collected from EUMCSH.

Final diagnoses for each participant were classified based on medical history, questionnaires, and PSG using the following criteria: OSA was defined as an Apnea-Hypopnea Index (AHI)≥15 per hour, and an ISI<15. Insomnia was defined as an AHI<15 per hour and ISI≥15. In cases of COMISA, the AHI was ≥15 per hour, and the ISI was ≥15. Finally, a negative group was defined as having an AHI<15 per hour, and an ISI<15. Since the sensitivity of OSA screening questionnaires is higher for moderate to severe OSA (AHI of 15 or more) than for mild OSA (AHI of 15 or less) [25], we used AHI≥15 per hour as the cut-off for OSA in this study.

### Measures

#### Overview

As part of the study protocol, subjects completed a series of self-report questionnaires prior to undergoing PSG. Basic demographic characteristics such as age, sex, height, weight, and BMI were also collected. For the SMC data set, marital status, education, occupation, neck, waist, and hip circumference were additionally recorded. The participants displayed diversity in their age distribution, marital status, occupation, and level of education.

#### Insomnia Severity Index

The subjective severity of insomnia symptoms during the past 2 weeks was measured with the Korean version of the ISI [39]. The ISI consists of 7 items, and each item was measured from 0 (no symptom) to 4 (very severe). The final score ranges from 0 to 28 points, with higher scores indicating more severe insomnia [27]. Subjects with ISI>14 are considered to have clinical insomnia.

#### Epworth Sleepiness Scale

Daytime sleepiness was measured with the Korean version of the ESS [40] translated from the ESS [28]. The ESS consists of 8 items, and each item was measured on a 0 (no napping) to 3 (high chance for napping) scale, with total scores ranging from 0 to 24. Subjects with an ESS>10 are considered to have excessive daytime sleepiness.

#### Pittsburgh Sleep Quality Index

The Korean version of the PSQI questionnaire measures the subjective evaluation of sleep quality during the past month and consists of 18 items [41]. PSQI has 7 components: sleep quality, sleep onset latency, sleep duration, sleep efficiency, sleep disturbance, use of sleeping medication, and daytime dysfunction [29]. The score of each question is 0-3. The total score of the PSQI is from 0 to 21, with higher scores indicating poorer sleep quality.

#### Polysomnography

PSG measurements were recorded during one night of observation with standard electrodes and sensors using Embla N7000 (Medcare-Embla). PSG data were evaluated according to the manual of the American Academy of Sleep Medicine [42].

### Model Building

In this work, we used XGBoost for classification. XGBoost is a tree-based machine learning algorithm using gradient boosting [36] and has been widely used in medical diagnosis because of its high accuracy and performance, straightforward interpretation, and ability to handle missing values [43-47]. Logistic regression (LR), random forest (RF), and support vector classifier were also implemented for comparison.

The SMC data set was randomly divided into training and testing sets in a ratio of 8:2 to develop a model. To deal with the imbalanced data, we used stratified splitting to maintain the ratio of positive values for the training and testing sets. We used stratified 5-fold cross-validation to train the XGBoost model, dividing the training set into 5-folds and training the model using 4-folds while testing on the remaining fold. We used Bayesian optimization to choose hyperparameters that maximize the average target function of the 5-fold cross-validation [48]. The XGBoost model was tuned based on max_depth, learning_rate, n_estimators, gamma, min_child_weight, max_delta_step, subsample, and colsample_bytree, and the ranges of the values were 2-10, 0.001-0.3, 50-4000, 0.01-1, 1-10, 0-0.1, 0.7-0.8, and 0.5-0.99, respectively. For OSA, the AUROC value was used as the target function of the model. For COMISA and insomnia, since the labels were imbalanced, we chose the *F*_1_ score as a target function, which tends to perform better than the AUROC for imbalanced data [49]. In addition, we gave different weights to positive and negative classes (proportional to the inverse of the class size) to prevent the model from ignoring the minority class during training.

### Feature Selection

To identify important features, we built the XGBoost model using the SMC data set and the SHAP method [38]. SHAP is a game theoretic method that explains the prediction of the model by calculating the impact of each feature on the model output. Positive (negative) SHAP values indicate a positive (negative) effect on the model’s output. Feature importance can be calculated by averaging the absolute SHAP values for all data.

### Statistical Analysis

We used AUROC and the area under the precision-recall curve (AUPRC) to evaluate the performance of the classification model. The receiver operating characteristics (ROC) curve plots the false-positive rate (the fraction of nonpatients mispredicted as a patient among all nonpatients) and true-positive rate (the fraction of correctly inferred patients among all predicted patients) as the probability threshold varies. The AUROC value (a value between 0 and 1) indicates the probability that the prediction model will successfully assign a higher risk value to a random positive participant than a random negative participant. When the data are imbalanced, AUPRC has been used to quantify the model performance [50]. A precision-recall curve plots precision (the fraction of correctly inferred patients among all predicted patients) and recall (true positive rate) as the probability threshold varies. Since the labels for COMISA and insomnia are imbalanced, we also use the AUPRC value to evaluate the performance of our model. We also provide the 95% CIs of the AUROC and AUPRC values on both training sets, which were estimated using bootstrapping (n=10,000). XGBoost and other classification models were implemented in Python (version 3.8) using Scikit-Learn [51] and XGBoost [36] packages. Bayesian optimization was implemented using BayesianOptimization packages [52].

### Website Development

The website was built using React JS (Meta Open Source) for the front end and Django (Django Software Foundation) for the back end. React JS provides an intuitive and easy-to-use interface through rich JS libraries, while Django allows for easy connection of the model with the back-end server using Python. The web app connects with the model through the Django server, which loads it directly from a checkpoint. The website is designed to be easy to use and allows users to predict their risk of having OSA, COMISA, and insomnia and to see how that risk changes with changes in weight or age. It includes (1) questionnaires for the user to fill out, (2) a prediction of the risk of having a sleep disorder, (3) a visualization of how risk changes with changes in weight or age, and (4) a description of each sleep disorder. Since XGBoost is a stochastic model, the results may vary with different random seeds. To make a robust prediction, we integrated the output from 10 models by changing the random seed when providing a change in risk with varying weight or age. Using 10 different results, we provided the upper and lower CIs as well as the mean prediction.

## Results

### Data Description

The demographic and clinical characteristics of the SMC data set are listed in Table 1. The average age was 50.5 (SD 14.1) years, and the average BMI was 25.8 (SD 4.2) kg/m^2^. According to diagnosis, the average age of the COMISA group was 55.0 (SD 12.9) years; for the OSA group it was 52.1 (SD 13.0) years, the insomnia group 50.0 (SD 13.8) years, and the negative group 43.0 (SD 14.9) years. The proportion of males was the highest in the OSA group at 86% (1770/2059), followed by the COMISA group at 77.1% (623/808), the negative group at 62.1% (577/929), and the insomnia group at 41.4% (191/461).

**Table 1:** Demographic and clinical characteristics of the SMC^a^ data set (n=4257).

Characteristics		Total	OSA^b^ (n=2059)	Insomnia (n=461)	COMISA^c^ (n=808)	Negative (n=929)
**Age (years), mean (SD)**		50.5 (14.1)	52.1 (13.0)	50.0 (13.8)	55.0 (12.9)	43.0 (14.9)
	<30, n (%)	392 (9.2)	105 (5.1)	49 (10.6)	35 (4.3)	203 (21.9)
	30s, n (%)	637 (15)	283 (13.7)	51 (11.1)	79 (9.8)	224 (24.1)
	40s, n (%)	802 (18.8)	398 (19.3)	102 (22.1)	127 (15.7)	175 (18.8)
	50s, n (%)	1234 (29.0)	657 (31.9)	147 (31.9)	247 (30.6)	183 (19.7)
	60s, n (%)	883 (20.7)	453 (22.0)	87 (18.9)	234 (29.0)	109 (11.7)
	≥70, n (%)	309 (7.3)	163 (7.9)	25 (5.4)	86 (10.6)	35 (3.8)
Male, n (%)		3161 (74.3)	1770 (86.0)	191 (41.4)	623 (77.1)	577 (62.1)
**Marital status, n (%)**						
	Single	863 (20.3)	332 (16.1)	101 (21.9)	122 (15.1)	308 (33.2)
	Married	2994 (70.3)	1560 (75.8)	305 (66.2)	588 (72.8)	541 (58.2)
	Divorced	196 (4.6)	88 (4.3)	30 (6.5)	47 (5.8)	31 (3.3)
	Widowed	106 (2.5)	41 (2.0)	14 (3.0)	31 (3.8)	20 (2.2)
	Unknown	98 (2.3)	38 (1.8)	11 (2.4)	20 (2.5)	29 (3.1)
**Education (years), n (%)**						
	≤6	116 (2.7)	48 (2.3)	9 (2.0)	48 (5.9)	11 (1.2)
	~7-12	1061 (24.9)	486 (23.6)	149 (32.3)	252 (31.2)	174 (18.7)
	~13-16	2095 (49.2)	1029 (50.0)	221 (47.9)	346 (42.8)	499 (53.7)
	>16	882 (20.7)	457 (22.2)	73 (15.8)	150 (18.6)	202 (21.7)
	Unknown	103 (2.4)	39 (1.9)	9 (2.0)	12 (1.5)	43 (4.6)
**Occupation, n (%)**						
	Yes	3021 (71)	1587 (77.1)	256 (55.5)	553 (68.4)	625 (67.3)
	Student	164 (3.9)	52 (2.5)	10 (2.2)	15 (1.9)	87 (9.4)
	No	968 (22.7)	379 (18.4)	185 (40.1)	220 (27.2)	184 (19.8)
	Unknown	104 (2.4)	41 (2)	10 (2.2)	20 (2.5)	33 (3.6)
**Anthropometric data, mean (SD)**						
	Height (cm)	168.0 (8.7)	169.4 (8.0)	163.0 (9.2)	167.5 (8.8)	167.9 (9.0)
	Weight (kg)	73.3 (15.1)	77.2 (14.4)	63.2 (12.7)	75.4 (15.6)	67.7 (13.3)
	BMI (kg/m^2^)	25.8 (4.2)	26.8 (4.1)	23.6 (3.7)	26.8 (4.5)	23.9 (3.5)
	NC^d^ (cm)	38.1 (4.0)	39.3 (3.4)	35.0 (3.5)	39.0 (3.8)	36.0 (3.7)
	WC^e^ (cm)	91.0 (11.4)	94.1 (10.3)	83.7 (10.0)	94.2 (11.6)	84.9 (9.8)
	HC^f^ (cm)	96.8 (7.7)	98.2 (7.4)	93.0 (7.1)	97.6 (8.4)	94.6 (7.0)
**Overnight polysomnography, mean (SD)**						
	TIB^g^ (min)	427.4 (52.8)	423.7 (52.6)	442.9 (46.6)	424.5 (55.8)	430.5 (51.9)
	TST^h^ (min)	353.8 (65.2)	349.7 (64.4)	362.1 (60.2)	337.8 (69.9)	372.7 (60.1)
	AHI^i^ (per h)	30.1 (24.2)	41.8 (21.3)	6.2 (4.3)	40.5 (22.2)	6.9 (4.6)
**Sleep questionnaires, mean (SD)**						
	ISI^j^	11.4 (6.1)	8.2 (3.8)	19.2 (3.2)	18.5 (3.1)	8.2 (4.1)
	ESS^k^	9.7 (4.8)	9.6 (4.5)	9.2 (5.2)	11.0 (5.5)	9.0 (4.3)
	PSQI^l^	7.8 (3.8)	6.2 (2.7)	12.4 (3.6)	10.8 (3.5)	6.5 (3.0)

^a^SMC: Samsung Medical Center.

^b^OSA: obstructive sleep apnea.

^c^COMISA: comorbid insomnia and sleep apnea.

^d^NC: neck circumference.

^e^WC: waist circumference.

^f^HC: hip circumference.

^g^TIB: time in bed.

^h^TST: total sleep time.

^i^AHI: Apnea-Hypopnea Index.

^j^ISI: Insomnia Severity Index.

^k^ESS: Epworth Sleepiness Scale.

^l^PSQI: Pittsburgh Sleep Quality Index.

The demographic and clinical characteristics of the EUMCSH data set are listed in Table 2. The average age was 57.6 (SD 14.7) years and the average BMI was 26.4 (SD 4.6) kg/m^2^. According to diagnosis, the average age of the OSA group was 59.3 (SD 14.1) years; for the COMISA group it was 58.7 (SD 13.3) years, the insomnia group 49.7 (SD 16.2) years, and the negative group 49.2 (SD 16.5) years. The proportion of males was highest in the OSA group at 70.5% (146/207), followed by the COMISA group at 55% (55/100), the negative group at 40% (14/35), and the insomnia group at 39.1% (9/23).

**Table 2:** Demographic and clinical characteristics of the EUMCSH^a^ data set (n=365)^a^.

Characteristics			Total		OSA^b^ (n=207), mean (SD)		Insomnia (n=23), mean (SD)		COMISA^c^ (n=100), mean (SD)		Negative (n=35), mean (SD)
Age (years)			57.6 (14.7)		59.3 (14.1)		49.7 (16.2)		58.7 (13.3)		49.2 (16.5)
Male			224 (61.4)		146 (70.5)		9 (39.1)		55 (55.0)		14 (40.0)
**Anthropometric data**											
	Height (cm)	165.4 (9.4)		166.4 (9.5)		163.9 (8.9)		163.3 (9.5)		166.7 (7.9)	
	Weight (kg)	72.7 (15.9)		75.0 (15.5)		65.4 (14.0)		71.3 (17.3)		68.2 (12.2)	
	BMI (kg/m^2^)	26.4 (4.6)		26.9 (4.5)		24.2 (3.6)		26.6 (5.2)		24.4 (3.1)	
**Items of ISI^d^**											
	ISI 1a	1.3 (1.3)		0.7 (0.9)		2.3 (1.3)		2.7 (1.1)		0.8 (0.9)	
	ISI 1b	1.5 (1.3)		0.9 (0.9)		2.5 (1.3)		2.8 (1.0)		1.1 (1.0)	
	ISI 1c	1.8 (1.3)		1.3 (1.1)		2.3 (1.5)		2.9 (1.0)		1.5 (1.0)	
	ISI 2	2.4 (1.3)		1.8 (1.1)		3.3 (1.1)		3.6 (0.7)		2.1 (1.1)	
	ISI 5	1.6 (1.5)		0.8 (1.0)		3.2 (1.0)		3.1 (0.9)		1.0 (1.3)	

^a^EUMCSH: Ewha Womans University Medical Center Seoul Hospital.

^b^OSA: obstructive sleep apnea.

^c^COMISA: comorbid insomnia and sleep apnea.

^d^ISI: Insomnia Severity Index.

### Feature Selection

**Figure 2:**
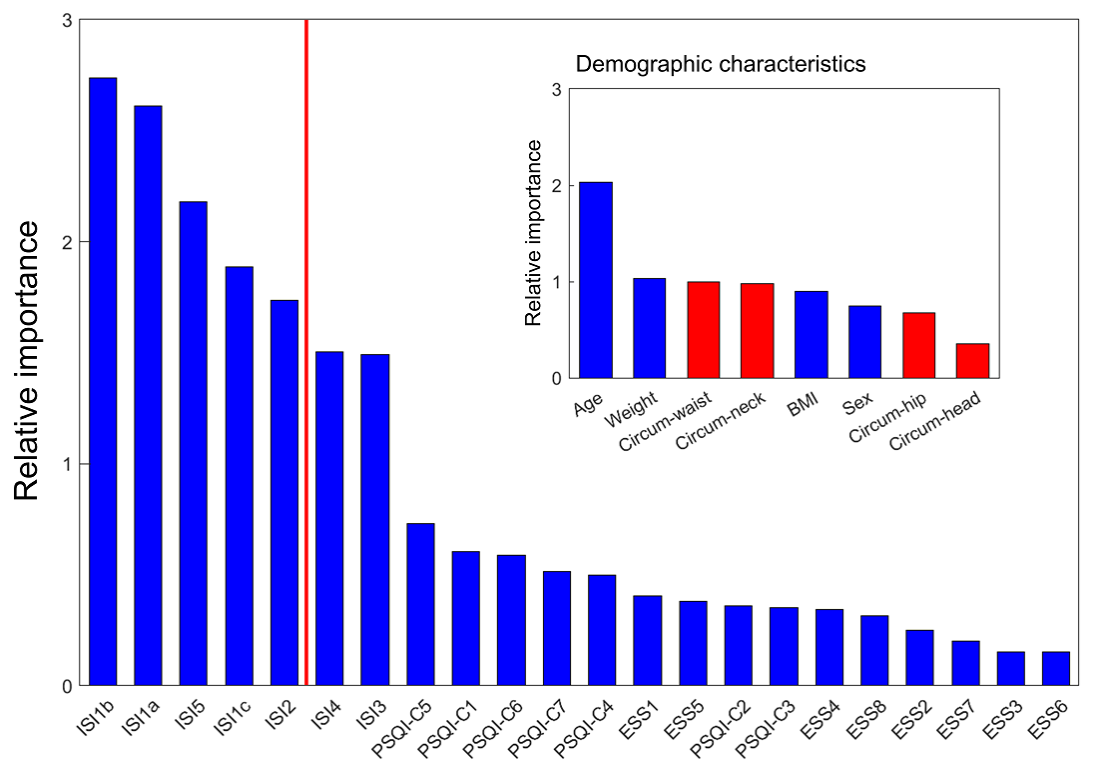
Selection of key features to predict the risk of sleep disorders. The importance of each feature is defined as the sum of the average of the absolute SHAP values in predicting OSA, COMISA, and insomnia. Among 22 items from sleep-related questionnaires, the top 5 items (ISI1b, ISI1a, ISI5, ISI1c, and ISI2) were selected. Among 8 demographic characteristics, 4 characteristics (age, BMI, weight, and sex), which can be easily answered, were selected (inset). ESS: Epworth Sleepiness Scale; ISI: Insomnia Severity Index; PSQI: Pittsburgh Sleep Quality Index; SHAP: Shapley additive explanations.

We aim to develop an algorithm that can accurately predict the risk of 3 sleep disorders—OSA, COMISA, and insomnia—using a simple set of questions selected from 22 items from sleep-related questionnaires and 8 demographic characteristics. To achieve this, we first built 3 XGBoost models using 30 input features to predict participants’ risk of having OSA, COMISA, and insomnia with the SMC training set. We then calculated the importance of these 30 features using the SHAP method. To identify the key features for predicting all 3 disorders simultaneously, we summed the feature importance for each of the sleep disorders (Figure 2). Then, we selected the top 5 items from the sleep-related questionnaires (ISI1b, ISI1a, ISI5, ISI1c, and ISI2) whose feature importance is greater than 1.5. Using the same threshold, age was selected among the demographic characteristics. Additionally, we selected 3 other characteristics that can be easily answered (weight, BMI, and sex) from a group of 6 characteristics (sex, weight, BMI, neck circumference, waist circumference, and hip circumference) with feature importance values close to 1. In total, we used 9 features (5 items from the sleep-related questionnaires and 4 demographic characteristics) to develop a simple questionnaire predicting sleep disorders (SLEEPS) based on 3 XGBoost models trained using the SMC training set to predict the risk of OSA, COMISA, and insomnia.

### Results of Model Performance

**Figure 3:**
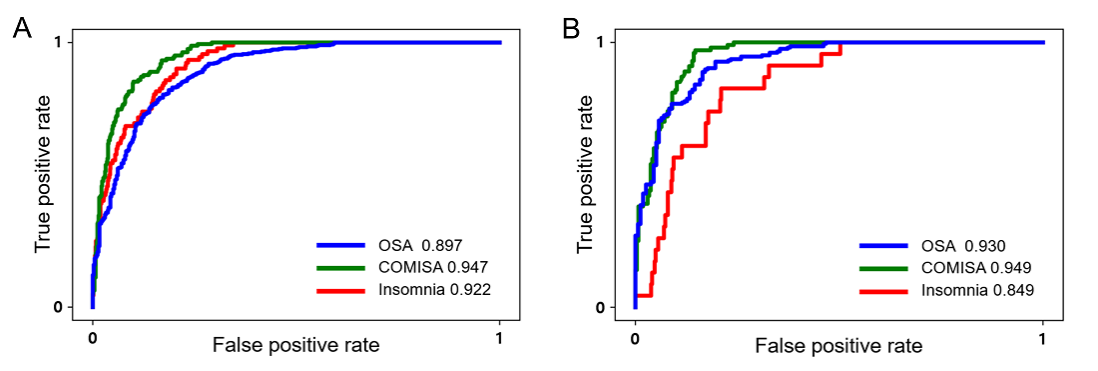
ROC curve of SLEEPS applied to the SMC testing set and EUMCSH data set. The ROC curve of the XGBoost model in the SMC testing set (A) and EUMCSH data set (B). The numbers denote the AUROC value. AUROC: area under receiver operating characteristic; COMISA: comorbid insomnia and sleep apnea; EUMCSH: Ewha Womans University Medical Center Seoul Hospital; OSA: obstructive sleep apnea; ROC: receiver operating characteristic; SMC: Samsung Medical Center; XGBoost: extreme gradient boosting.

We tested the performance of SLEEPS using the SMC testing set (Table 3 and Figure 3A). SLEEPS has high accuracy in predicting the risk of OSA (AUROC=0.897), COMISA (AUROC=0.947), and insomnia (AUROC=0.922). The AUPRC values for each disorder are as follows: OSA (0.877), COMISA (0.786), and insomnia (0.611). Note that SLEEPS outperformed the recently developed OSA screening model BASH-GN [29] (AUROC=0.78 and AUPRC=0.76), although BASH-GN outperformed the commonly used OSA screening questionnaires such as STOP-BANG [25] and Berlin [26].

**Table 3:** Mean and 95% CI of AUROC^a^ and AUPRC^b^ values of SLEEPS^c^ in the SMC^d^ testing set and EUMCSH^e^ data set.

Performance		AUROC (95% CI)	AUPRC (95% CI)	AUPRC baseline
**SMC testing set (n=851)**				
	OSA^f^	0.897 (0.883-0.916)	0.877 (0.850-0.901)	0.484
	COMISA^g^	0.947 (0.940-0.962)	0.786 (0.727-0.836)	0.190
	Insomnia	0.922 (0.915-0.946)	0.611 (0.521-0.693)	0.108
**EUMCSH data set (n=365)**				
	OSA	0.930 (0.903-0.954)	0.940 (0.911-0.954)	0.567
	COMISA	0.949 (0.927-0.968)	0.854 (0.780-0.913)	0.274
	Insomnia	0.849 (0.784-0.905)	0.235 (0.119-0.379)	0.063

^a^AUROC: area under the receiver operating characteristics.

^b^AUPRC: area under the precision-recall curve.

^c^SLEEPS: simple questionnaire predicting sleep disorders.

^d^SMC: Samsung Medical Center.

^e^EUMCSH: Ewha Womans University Medical Center Seoul Hospital.

^f^OSA: obstructive sleep apnea.

^g^COMISA: comorbid insomnia and sleep apnea.

The AUROC values for all 3 sleep disorders are greater than 0.897, indicating that SLEEPS can accurately predict the risk of these disorders (Figure 3A). The seemingly low AUPRC values for COMISA and insomnia are due to the imbalance in the data, with a higher proportion of negative cases. To account for this, we compared the AUPRC values to the AUPRC baseline, which is the proportion of positive cases in the data. The OSA label is balanced, with a similar proportion of positive and negative cases, and SLEEPS has a high AUPRC value for predicting OSA (0.877). Despite the imbalanced labels for insomnia and COMISA (AUPRC baselines of 0.108 and 0.190, respectively), SLEEPS still has high AUPRC values (0.611 and 0.786, respectively), demonstrating its ability to distinguish between those with and without these disorders even with imbalanced data.

To evaluate the generalizability of SLEEPS, we also tested it using the EUMCSH data set as an independent test set. SLEEPS maintained similar performance on this new data set (Figure 3B and Table 3), showing that it is scalable and not dependent on specific data. In particular, the AUPRC values for OSA and COMISA increased for the EUMCSH data set, while the AUPRC value for insomnia decreased due to the more imbalanced data in the EUMCSH data set (AUPRC baseline=0.063). To investigate the low AUPRC value in predicting insomnia within the EUMCSH data set, we examined the model’s performance using a balanced subset of the data. We randomly sampled non-insomnia participants from the EUMCSH data set to match the number of insomnia participants, thus creating a perfectly balanced data set. Subsequently, we calculated the AUPRC value of SLEEPS on this balanced data. This process was repeated 10,000 times, and the results consistently showed higher AUPRC values compared to using the entire EUMCSH data set (Table S1 in Multimedia Appendix 1). This suggests that the low AUPRC value for predicting insomnia in the EUMCSH data set is primarily attributable to the high data imbalance. Furthermore, the SLEEPS model demonstrates high performance when applied to a balanced data set.

In contrast to previous OSA screening studies [25,26,30,31,33,35], we separated COMISA from OSA. To investigate the importance of this separation, we merged COMISA with OSA to create a new OSA label and retrained the XGBoost model predicting this label using the SMC training set. We then evaluated the model’s performance on the SMC testing set and EUMCSH data set (Table S2 in Multimedia Appendix 1). Interestingly, for both testing sets, the AUROC and AUPRC values of the model decreased for merged OSA, emphasizing that distinguishing COMISA from OSA is critical for accurate prediction. On the other hand, when we merged COMISA with insomnia and retrained the model, the prediction performance for insomnia increased for both testing sets, indicating that it is challenging to distinguish COMISA from insomnia.

We also examined if the performance of SLEEPS could be improved by adding additional features. We had not previously included neck, waist, and hip circumferences as input features because they are not easily measurable (Figure 2 inset). Thus, we created a new XGBoost model that considers these 3 features in addition to the 9 previously selected features. Even after adding the 3 input features, there is no significant improvement in prediction (Table S3 in Multimedia Appendix 1), indicating that the addition of these features did not significantly improve the model.

Furthermore, we also verified that XGBoost was the best choice for our classification model by developing and testing 3 other popular models: LR, RF, and support vector classifier. XGBoost outperformed the other models for all 3 sleep disorders on both the SMC testing set and the EUMCSH data set (Figures S1 and S2 and Tables S3 and S4 in Multimedia Appendix 1). In particular, the AUPRC for COMISA and insomnia was relatively higher for XGBoost than for the other 3 models, indicating that SLEEPS can accurately predict the risk even when the data are imbalanced.

### Impact of Each Feature Toward Prediction

**Figure 4:**
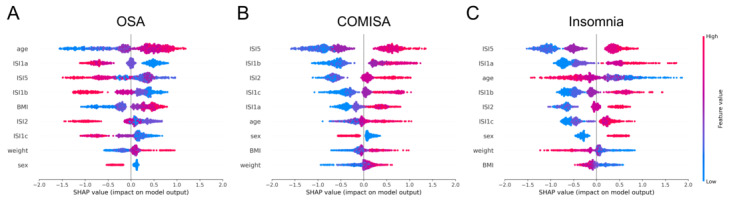
Impact of each feature on predicting sleep disorders. Beeswarm plot indicating how the features impact the output of SLEEPS predicting (A) OSA, (B) COMISA, and (C) insomnia on the SMC testing set. For each sleep disorder, the 9 features are ordered by their importance. Each point represents a single participant, and its color represents the SHAP value, with a range from blue to red, denoting low to high values. The sign of the x-axis indicates the direction of the contribution: positive values indicate a positive contribution to the output (risk of having a sleep disorder). COMISA: comorbid insomnia and sleep apnea; ISI: Insomnia Severity Index; OSA: obstructive sleep apnea; SHAP: Shapley additive explanations; SLEEPS: simple questionnaire predicting sleep disorders; SMC: Samsung Medical Center.

To understand how SLEEPS differentiates between individuals with sleep disorders and those without, we calculated the SHAP values of the 9 features used in the model. A positive (negative) SHAP value indicates that the feature has a positive (negative) impact on the model’s output. We visualized the distribution of SHAP values for each feature using a beeswarm plot, where each dot corresponds to one participant and its color represents the SHAP value, with blue corresponding to low values and red corresponding to high values (Figure 4). For example, males (encoded as 0, blue dots) tend to have a higher chance of having OSA and COMISA than females (encoded as 1, red dots; Figure 4A and B). However, females have higher chances of having insomnia than men (Figure 4C). This is consistent with a previous study showing that females generally report having a greater sleep need and more frequently report poor or insufficient sleep than males [53]. A meta-analysis study also reported that the prevalence of insomnia is approximately 1.41 times more common in females than males [54]. Overall, the demographic characteristics (age, sex, weight, and BMI) have similar effects on OSA and COMISA, but opposite effects on insomnia. On the other hand, the questionnaire items (ISI1a, ISI1b, ISI1c, ISI2, and ISI5) have similar effects on COMISA and insomnia, but opposite effects on OSA (Figure 4). The beeswarm plot for the EUMCSH data set (Figure S3 in Multimedia Appendix 1) also shows a consistent tendency for each feature.

### Probability Prediction

**Figure 5:**
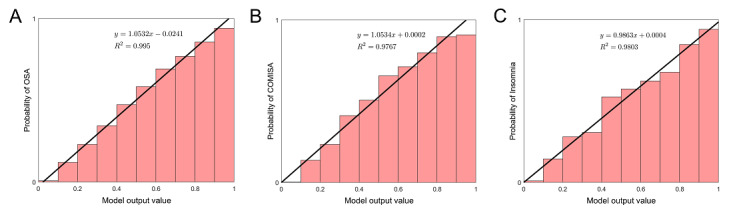
Probability prediction of each sleep disorder using SLEEPS. Histogram indicating the percentage of people in the SMC data set having (A) OSA, (B) COMISA, and (C) insomnia for the given model output range (posterior distribution). Black lines indicate linear regression. COMISA: comorbid insomnia and sleep apnea; OSA: obstructive sleep apnea; SLEEPS: simple questionnaire predicting sleep disorders; SMC: Samsung Medical Center.

In the case of COMISA and insomnia, the labels in the data are imbalanced. Therefore, it might be misleading to interpret the model output (a number between 0 and 1) directly as the “probability” or “risk” of having a sleep disorder. To verify this issue, we divided the range of model output [0,1] into 10 equal intervals and observed the distribution of model output values for participants with and without a sleep disorder, respectively (Figure S4 in Multimedia Appendix 1). For each interval, we calculated the percentage of participants with model output in that interval who had sleep disorders (Figure 5). We then used linear regression to approximate this distribution (black line in Figure 5). Surprisingly, the linear fitting has a high *R*^2^ value in all 3 cases. Since we can consider the entire SMC data set as the representable data of the “ground truth” (or likelihood) of Korean individuals, the *y*-value for specific model output can be interpreted as the “probability” of having a particular sleep disorder using the Bayes formula. Furthermore, all 3 regression lines are similar to *y*=*x*, indicating that the model output can be interpreted as the probability of having a particular sleep disorder.

### Quantitative Suggestion

SLEEPS allows us to calculate the probability of having each of the 3 sleep disorders. In addition to this, we can also provide specific guidelines related to the 3 sleep disorders. Out of the 4 demographic characteristics used in SLEEPS, weight can be easily changed, and age increases over time. By varying the age or weight while holding the values of other features constant, we can predict how the probability of having the 3 sleep disorders changes as age or weight varies. This can provide quantitative answers to questions such as “How much weight should I lose to reduce my risk of having OSA below 0.5?” or “How will my risk of having COMISA change as I age?”

### SLEEPS-Based Website

**Figure 6:**
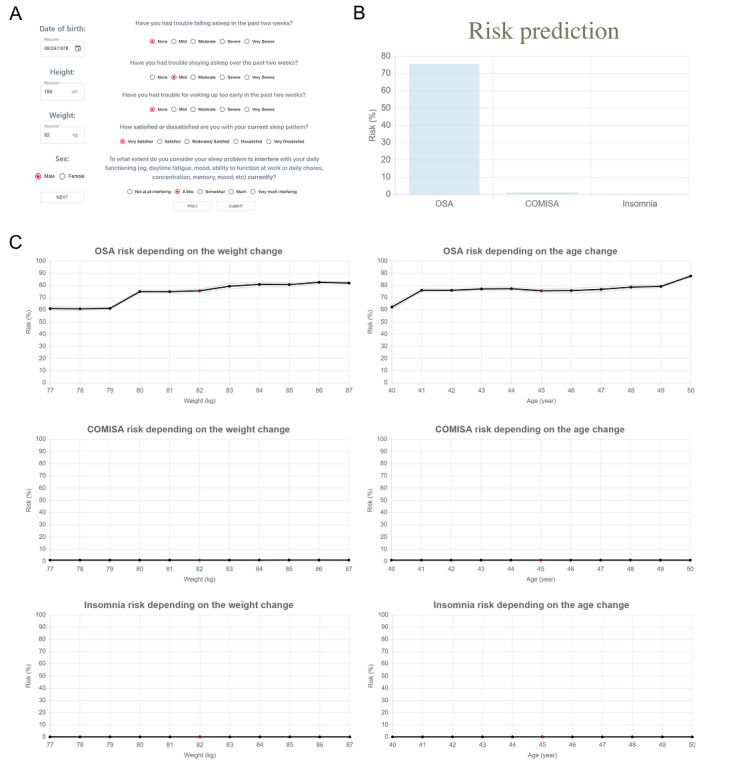
Screenshot of the website. Questions (A), results (B), and prediction with respect to weight change and age change provided by SLEEPS (C). For the graph, we used 10 different model outputs, by changing the random seed, to calculate the mean and the standard deviation of the prediction. COMISA: comorbid insomnia and sleep apnea; OSA: obstructive sleep apnea; SLEEPS: simple questionnaire predicting sleep disorders.

To make SLEEPS more widely available, we have also created a publicly available website [37] that predicts the risk of the 3 sleep disorders based on SLEEPS (Figure 6). When users input their answers to the 9 questions (Figure 6A), the website shows the predicted risk of the user having each of the 3 sleep disorders (Figure 6B). The website also provides a graph showing the predicted change in risk as weight varies or age increases (Figure 6C), along with the CIs for the prediction.

## Discussion

In this study, we developed SLEEPS, a 9-item questionnaire that can accurately predict the risk of OSA, COMISA, and insomnia simultaneously. Using SHAP values, the 5 items (ISI1b, ISI5, ISI1a, ISI1c, and ISI2) were selected among 22 items from sleep-related questionnaires and 4 characteristics (age, BMI, weight, and sex) were selected among 8 demographic characteristics. We used XGBoost to create a prediction model using these 9 features. Originally, the problem could be considered a multiclass classification problem, predicting the participants’ probability of having OSA, COMISA, insomnia, or no disorder. Since binary classifications can theoretically be used to represent a multiclass classification [55], we used 3 different XGBoost models to predict the 3 sleep disorders separately to simplify the problem.

The 9 features we used are easy to answer, unlike those in previously developed questionnaires [25,26,30,31,33,35], which require information such as observed breathing cessation and systolic and diastolic blood pressure. Despite using only simple items, SLEEPS showed high accuracy in predicting the risk of sleep disorders and outperformed previously developed OSA or insomnia screening questionnaires [34,35]. The use of XGBoost contributed to this improved performance, as other popular classification machine learning algorithms showed poorer performance (Tables S4 and S5 in Multimedia Appendix 1). This explains the superior performance of SLEEPS in comparison to previously developed OSA or insomnia screening questionnaires that were based on LR. Furthermore, we found that distinguishing COMISA from OSA is critical for accurate prediction (Table S2 in Multimedia Appendix 1), unlike previous studies [25,26,30,31,33,35] where COMISA and OSA were not distinguished. Specifically, the model’s performance decreased when merging the COMISA and OSA labels. This indicates that the COMISA group differs from the OSA group and must be distinguished for accurate risk prediction. In future work, it would be valuable to further investigate the distinct characteristics and underlying mechanisms that differentiate COMISA from OSA. This understanding could lead to the development of tailored interventions and personalized treatment strategies, ultimately improving patient outcomes in both COMISA and OSA populations.

We also found that older people, overweight people, and males tend to have a higher risk of OSA or COMISA, while younger people, slim people, and females are more likely to have insomnia. People who responded with high values to ISI questions have a higher probability of having insomnia and COMISA while having a lower tendency to have OSA. This is because people with both OSA and high ISI scores often have COMISA.

SLEEPS can be easily accessed via [37]. Importantly, our website provides predictions for the risk of the 3 sleep disorders based on an individual’s current and future characteristics, such as weight and age. This information can be useful in developing treatment plans for patients. For instance, OSA-diagnosed patients are usually advised to lose weight [2], but they may be more motivated if they understand the expected improvement in their OSA severities. SLEEPS can provide a quantitative suggestion for how much weight an individual should lose to reduce the risk of OSA below a certain threshold, such as 50%. In addition, as age is an important factor in OSA [56], knowing the change in risk as age increases can help with monitoring the progress of OSA. Furthermore, SLEEPS can predict changes in the risk of sleep disorders due to aging, which may help prevent the progression of conditions like Alzheimer disease (AD). AD biomarkers increase with age, and OSA is also associated with an increase in AD markers amyloid-β and tau measured in cerebrospinal fluid [57]. If OSA is predicted in advance and treated actively, it might help prevent the occurrence as well as aggravation of AD.

There are several limitations to address in this study. First, the current version of SLEEPS is based on data from Korean participants in the SMC and EUMCSH data sets, which limits its applicability to individuals of other races. To extend SLEEPS to other races, we have searched for public data sets, including PSG data from various races in the National Sleep Research Resource [58]. However, we have not yet been able to find data that includes both PSG with an AHI that meets our standards and questionnaires about sleep symptoms and daytime dysfunction. If such data were available, incorporating it into the SLEEPS framework would be important in future work to make it more widely applicable. Second, the retrospective nature of this study limited the collection of socioeconomic information, including variables such as income level. It would be valuable for future work to incorporate and analyze socioeconomic factors to provide a more holistic understanding of sleep disorders and their associations. Furthermore, since our research aims to develop a screening model for predicting OSA, COMISA, and insomnia, we did not specifically emphasize genetic groups or their predispositions. However, incorporating additional information helping to identify phenotypes of patients or genetic groups with different predispositions to OSA, COMISA, and insomnia could impact the pretest probability and enhance the practicality and clinical value of our algorithm. Last, in this study, we used AHI≥15 per hour as the cut-off for OSA and did not consider mild OSA. However, the COMISA group exhibited a lower AHI than the OSA-only group when using an AHI threshold of 5 [16], indicating that many people with mild OSA experience insomnia symptoms. Therefore, developing a screening tool that can distinguish this specific subgroup would be important in future work.

In conclusion, we developed an XGBoost model SLEEPS which effectively predicts the risk of OSA, COMISA, and insomnia. By developing a publicly available website using this algorithm, we have created an easily accessible and user-friendly tool for evaluating the risk of sleep disorders, potentially enhancing the diagnosis and treatment process by offering greater accessibility and convenience.

## Appendix

Multimedia Appendix 1 Supplementary tables and figures.

## Abbreviations

AD: Alzheimer disease
AHI: Apnea-Hypopnea Index
AUPRC: area under the precision-recall curve
AUROC: area under the receiver operating characteristics
COMISA: comorbid insomnia and sleep apnea
ESS: Epworth Sleepiness Scale
EUMCSH: Ewha Womans University Medical Center Seoul Hospital
ISI: Insomnia Severity Index
LR: logistic regression
OSA: obstructive sleep apnea
PSG: polysomnography
PSQI: Pittsburgh Sleep Quality Index
RF: random forest
ROC: receiver operating characteristics
SHAP: shapley additive explanations
SLEEPS: simple questionnaire predicting sleep disorders
SMC: Samsung Medical Center
XGBoost: extreme gradient boosting

## Data Availability

The data sets analyzed during this study are available from the corresponding author upon reasonable request.
