# Supplementary material for: Predicting the Risk of Sleep Disorders Using a Machine Learning–Based Simple Questionnaire: Development and Validation Study
Source: J Med Internet Res. 2023 Sep 21;25:e46520. doi: 10.2196/46520 (PMC10557018; doi:10.2196/46520)

**Table S1.** Performance of simple questionnaire predicting sleep disorders (SLEEPS) on balanced and imbalanced insomnia datasets.

| Performance | AUROC^a^ (95% CI) | AUPRC^b^ (95% CI) | AUPRC baseline |
| --- | --- | --- | --- |
| EUMCSH^c^ dataset (n=365) | 0.849 (0.784-0.905) | 0.235 (0.119-0.379) | 0.063 |
| Part of EUMCSH dataset (n=46) | 0.862 (0.758-0.945) | 0.816 (0.665-0.946) | 0.5 |

^a^AUROC: area under the receiver operating characteristics.

^b^AUPRC: area under the precision-recall curve.

^c^EUMCSH: Ewha Womans University Medical Center Seoul Hospital.

**Table S2.** Performance of eXtreme Gradient Boosting (XGBoost) model using merged label, disregarding COMISA.^a^

| Performance | | AUROC^b^ (95% CI) | AUPRC^c^ (95% CI) | AUPRC baseline |
| --- | --- | --- | --- | --- |
| **SMC^d^ testing set (n=851)** | | | | |
|  | OSA^e^ (OSA + COMISA^a^) | 0.789 (0.757-0.822) | 0.875 (0.845-0.902) | 0.673 |
|  | Insomnia (Insomnia+COMISA) | 0.983 (0.977-0.989) | 0.964 (0.948-0.902) | 0.298 |
| **EUMCSH^f^ dataset (n=365)** | | | | |
|  | OSA (OSA + COMISA) | 0.691 (0.617-0.76) | 0.917 (0.879-0.948) | 0.841 |
|  | Insomnia (Insomnia+COMISA) | 0.982 (0.971-0.991) | 0.970 (0.945-0.984) | 0.337 |

^a^COMISA: comorbid insomnia and sleep apnea.

^b^AUROC: area under the receiver operating characteristics.

^c^AUPRC: area under the precision-recall curve.

^d^SMC: Samsung Medical Center.

^e^OSA: obstructive sleep apnea.

^f^EUMCSH: Ewha Womans University Medical Center Seoul Hospital.

**Table S3.** Adding circumference information is not beneficial. Comparison between SLEEPS^a^ (using nine features) and the XGBoost^b^ model including circumference information of neck, waist, and hip as additional input features (total 12 features). In either case, the AUROC^c^ value was similar. Moreover, SLEEPS has a higher AUPRC^d^ value for COMISA^e^ and insomnia than the model with the circumference information.

| SMC^f^ testing (n=851) | | AUROC^c^ (95% CI) | AUPRC^d^ (95% CI) |
| --- | --- | --- | --- |
| **SLEEPS^a^ (9 input features)** | | | |
|  | OSA^g^ | 0.897 (0.883-0.916) | 0.877 (0.850-0.901) |
|  | COMISA^e^ | 0.947 (0.940-0.962) | 0.786 (0.727-0.836) |
|  | Insomnia | 0.922 (0.915-0.946) | 0.611 (0.521-0.693) |
| **XGBoost^b^ with 12 input features (Circumference information added)** | | | |
|  | OSA | 0.900 (0.877-0.917) | 0.882 (0.853-0.909) |
|  | COMISA | 0.946 (0.932-0.960) | 0.765 (0.709-0.842) |
|  | Insomnia | 0.918 (0.896-0.939) | 0.555 (0.443-0.658) |

^a^SLEEPS: simple questionnaire predicting sleep disorders.

^b^XGBoost: extreme gradient boosting.

^c^AUROC: area under the receiver operating characteristics.

^d^AUPRC: area under the precision-recall curve.

^e^COMISA: comorbid insomnia and sleep apnea.

^f^SMC: Samsung Medical Center.

^g^OSA: obstructive sleep apnea.

**Table S4.** Summary of the performance of 4 candidate algorithms on SMC^a^ testing set. The mean and 95% CI of the AUROC^b^ and AUPRC^c^ values of 4 candidate algorithms in the SMC testing set. The highest AUROC and AUPRC values are shown in bold. Number next to the name of the sleep disorder represents the ratio of positive class for each data set.

| SMC^a^ testing set (n=851) | | AUROC^b^ (95% CI) | AUPRC^c^ (95% CI) |
| --- | --- | --- | --- |
| **OSA^d^ (0.484)** | | | |
|  | LR^e^ | 0.872 (0.848-0.895) | 0.846 (0.809-0.895) |
|  | SVC^f^ | 0.847 (0.821-0.872) | 0.825 (0.787-0.859) |
|  | RF^g^ | 0.891 (0.868-0.911) | 0.866 (0.830-0.897) |
|  | XGBoost^h^ | 0.897 (0.883-0.916) | 0.877 (0.850-0.901) |
| **COMISA^i^ (0.190)** | | | |
|  | LR | 0.926 (0.907-0.943) | 0.682 (0.598-0.762) |
|  | SVC | 0.925 (0.906-0.942) | 0.729 (0.654-0.793) |
|  | RF | 0.940 (0.924-0.955) | 0.743 (0.662-0.817) |
|  | XGBoost | 0.947 (0.940-0.962) | 0.786 (0.727-0.836) |
| **Insomnia (0.108)** | | | |
|  | LR | 0.913 (0.889-0.935) | 0.563 (0.456-0.658) |
|  | SVC | 0.880 (0.848-0.909) | 0.514 (0.403-0.618) |
|  | RF | 0.895 (0.864-0.922) | 0.559 (0.446-0.661) |
|  | XGBoost | 0.922 (0.915-0.946) | 0.611 (0.521-0.693) |

^a^SMC: Samsung Medical Center.

^b^AUROC: area under the receiver operating characteristics.

^c^AUPRC: area under the precision-recall curve.

^d^OSA: obstructive sleep apnea.

^e^LR: logistic regression.

^f^SVC: support vector classifier.

^g^RF: random forest.

^h^XGBoost: extreme gradient boosting.

^i^COMISA: comorbid insomnia and sleep apnea.

**Table S5.** Summary of the performance of 4 candidate algorithms on EUMCSH^a^ dataset. The mean and 95% CI of the AUROC^b^ and AUPRC^c^ values of 4 candidate algorithms in the EUMCSH dataset. The highest AUROC and AUPRC values are shown in bold. Number next to the name of the sleep disorder represents the ratio of positive class for each data set.

| EUMCSH^a^ dataset (n=365) | | AUROC^b^ (95% CI) | AUPRC^c^ (95% CI) |
| --- | --- | --- | --- |
| **OSA^d^ (0.567)** | | | |
|  | LR^e^ | 0.894 (0.858-0.925) | 0.905 (0.863-0.939) |
|  | SVC^f^ | 0.887 (0.849-0.920) | 0.895 (0.851-0.931) |
|  | RF^g^ | 0.929 (0.900-0.954) | 0.929 (0.888-0.961) |
|  | XGBoost^h^ | 0.930 (0.903-0.954) | 0.940 (0.911-0.954) |
| **COMISA^i^ (0.274)** | | | |
|  | LR | 0.934 (0.908-0.956) | 0.818 (0.740-0.886) |
|  | SVC | 0.933 (0.907-0.955) | 0.801 (0.704-0.880) |
|  | RF | 0.941 (0.916-0.961) | 0.825 (0.732-0.896) |
|  | XGBoost | 0.949 (0.927-0.968) | 0.854 (0.780-0.913) |
| **Insomnia (0.063)** | | | |
|  | LR | 0.846 (0.779-0.897) | 0.219 (0.111-0.358) |
|  | SVC | 0.792 (0.715-0.960) | 0.159 (0.085-0.268) |
|  | RF | 0.822 (0.750-0.885) | 0.181 (0.100-0.290) |
|  | XGBoost | 0.849 (0.784-0.905) | 0.235 (0.119-0.379) |

^a^EUMCSH: Ewha Womans University Medical Center Seoul Hospital.

^b^AUROC: area under the receiver operating characteristics.

^c^AUPRC: area under the precision-recall curve.

^d^OSA: obstructive sleep apnea.

^e^LR: logistic regression.

^f^SVC: support vector classifier.

^g^RF: random forest.

^h^XGBoost: extreme gradient boosting.

^i^COMISA: comorbid insomnia and sleep apnea.

**Figure S1.** Performance evaluation of 4 candidate algorithms on SMC testing set. ROC curve (A,B,C) and PRC (D,E,F) of 4 machine learning models for OSA (A,D), COMISA (B,E), and insomnia (C,F) risk prediction on SMC testing data. COMISA: comorbid insomnia and sleep apnea; EUMCSH: Ewha Womans University Medical Center Seoul Hospital; LR: logistic regression; OSA: obstructive sleep apnea; PRC: precision-recall curve; RF: random forest; ROC: receiver operating characteristic; SMC: Samsung Medical Center; SVC: support vector classifier; XGBoost: extreme gradient boosting.


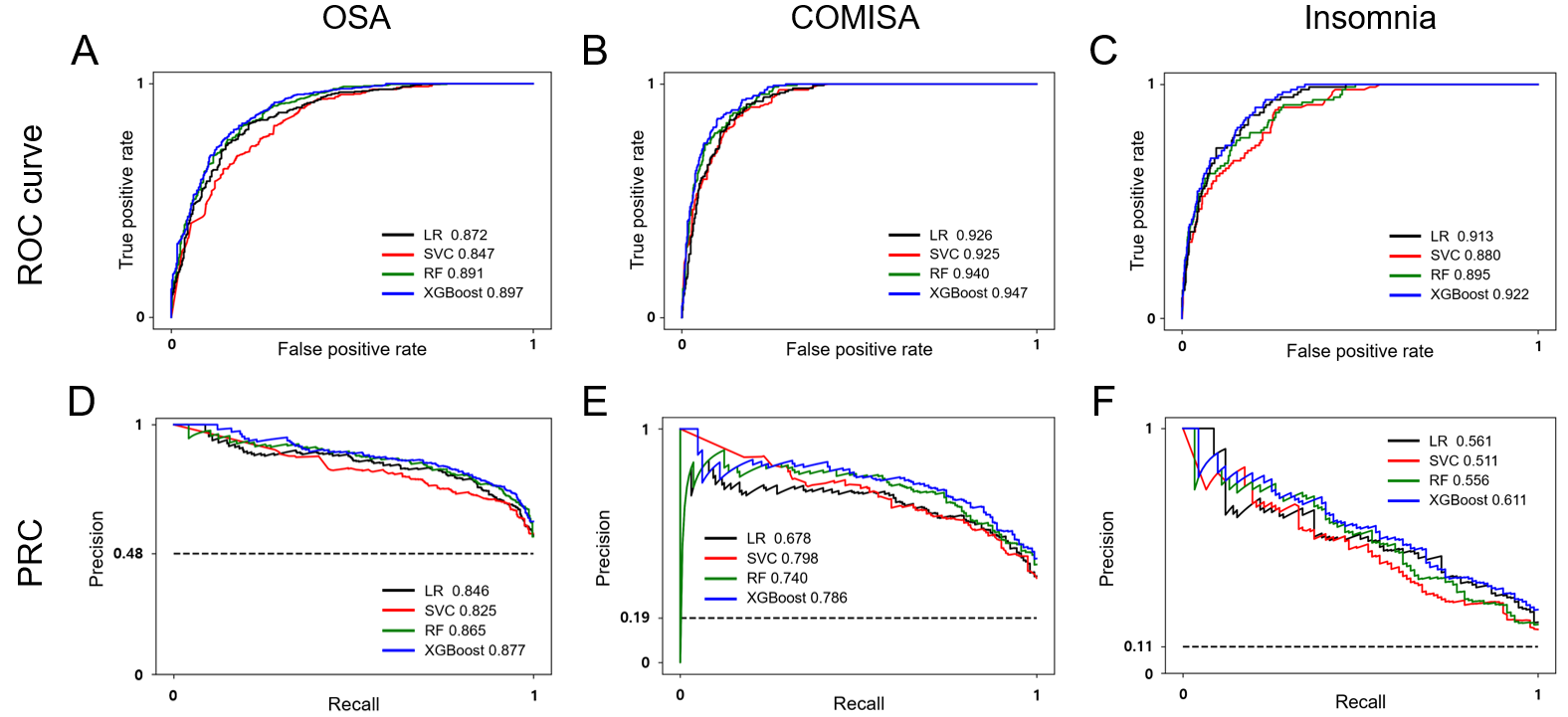


**Figure S2.** Performance evaluation of 4 candidate algorithms on EUMCSH dataset. ROC curve (A,B,C) and PRC (D,E,F) of 4 machine learning models for OSA (A,D), COMISA (B,E), and insomnia (C,F) risk prediction on EUMCSH dataset. COMISA: comorbid insomnia and sleep apnea; EUMCSH: Ewha Womans University Medical Center Seoul Hospital; LR: logistic regression; OSA: obstructive sleep apnea; PRC: precision-recall curve; RF: random forest; ROC: receiver operating characteristic; SMC: Samsung Medical Center; SVC: support vector classifier; XGBoost: extreme gradient boosting.


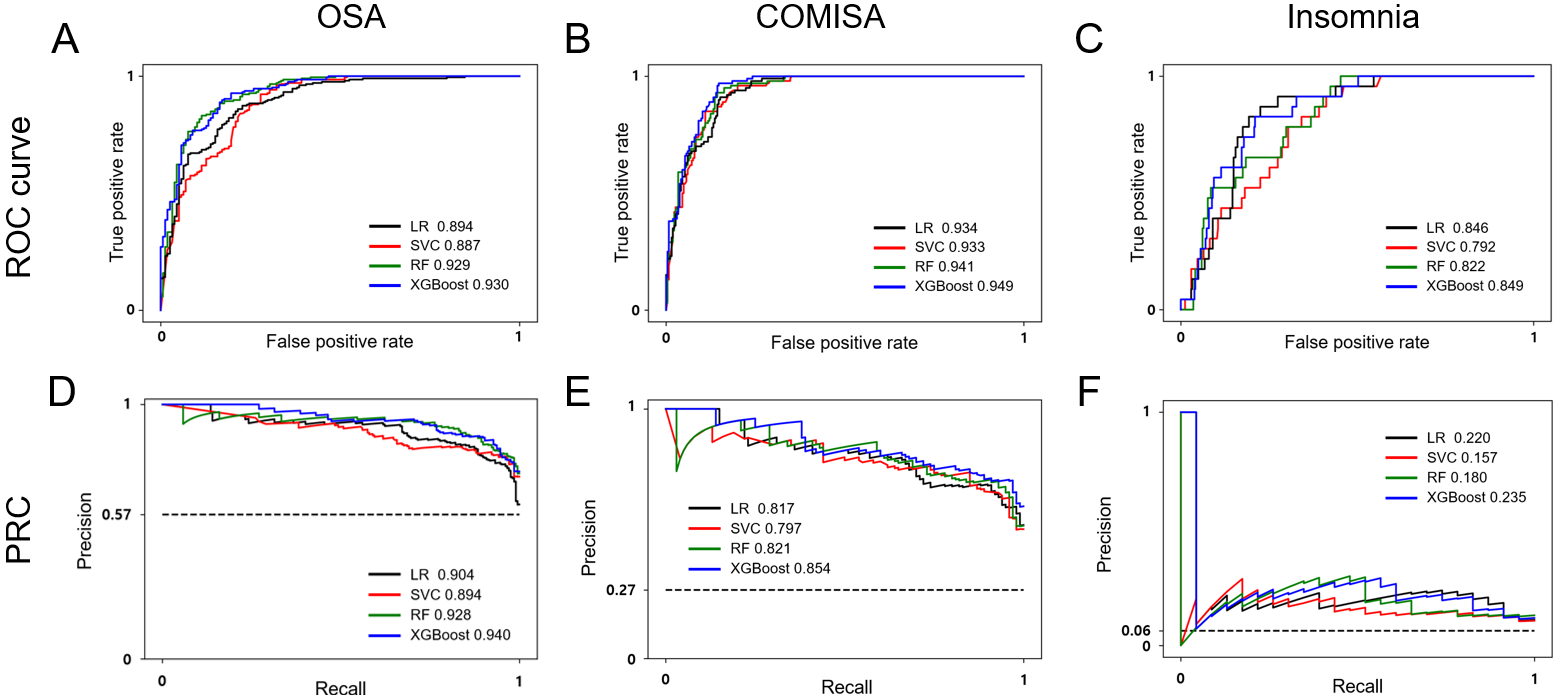


**Figure S3.** Impact of each feature on predicting sleep disorders. Beeswarm plot indicating how the features impact the output of SLEEPS predicting (A) OSA, (B) COMISA, and (C) insomnia on the EUMCSH dataset. For each graph, the 9 features are ordered by their importance. Each point represents a single participant, and its color represents the SHAP value, with a range from blue to red, denoting low to high values. The sign of the x-axis indicates the direction of the contribution: positive values indicate a positive contribution to the output (risk of having a sleep disorder). The tendency of each feature matches that of the SMC testing set. COMISA: comorbid insomnia and sleep apnea; EUMCSH: Ewha Womans University Medical Center Seoul Hospital; OSA: obstructive sleep apnea; SHAP: shapley additive explanations.

**
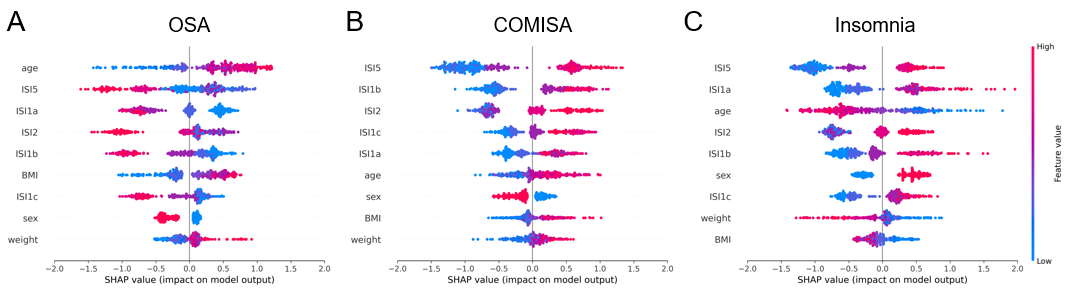
**

**Figure S4.** Distribution of the model output value of SLEEPS. The histogram indicates the model output of the SMC neurology clinic (A) OSA, (B) COMISA, and (C) insomnia. The distributions show separation between participants with (red histogram) and without (blue histogram) sleep disorders. COMISA: comorbid insomnia and sleep apnea; OSA: obstructive sleep apnea.


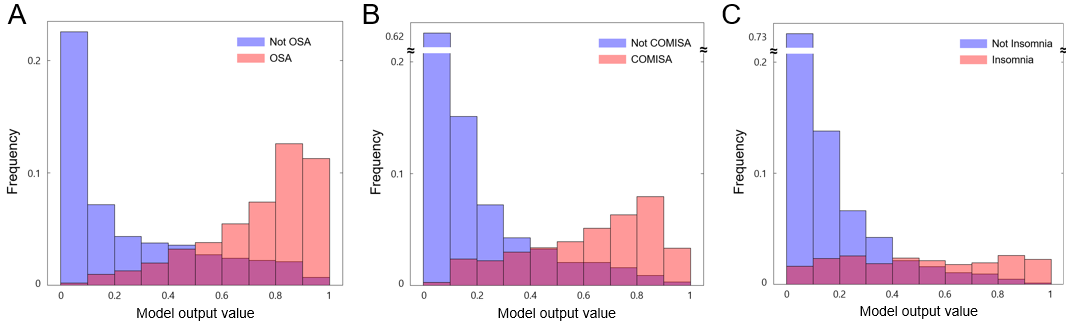

Supplement: Multimedia Appendix 1 [file jmir_v25i1e46520_app1.docx]
